# Correction: Periplasmic oxidized-protein repair during copper stress in *E*. *coli*: A focus on the metallochaperone CusF

**DOI:** 10.1371/journal.pgen.1010382

**Published:** 2022-09-01

**Authors:** The PLOS Genetics Staff

Fig 5 is incorrect. There is an error in panel A. The publisher apologizes for the error. Please see the correct Fig 5 here.

**Fig 5:**
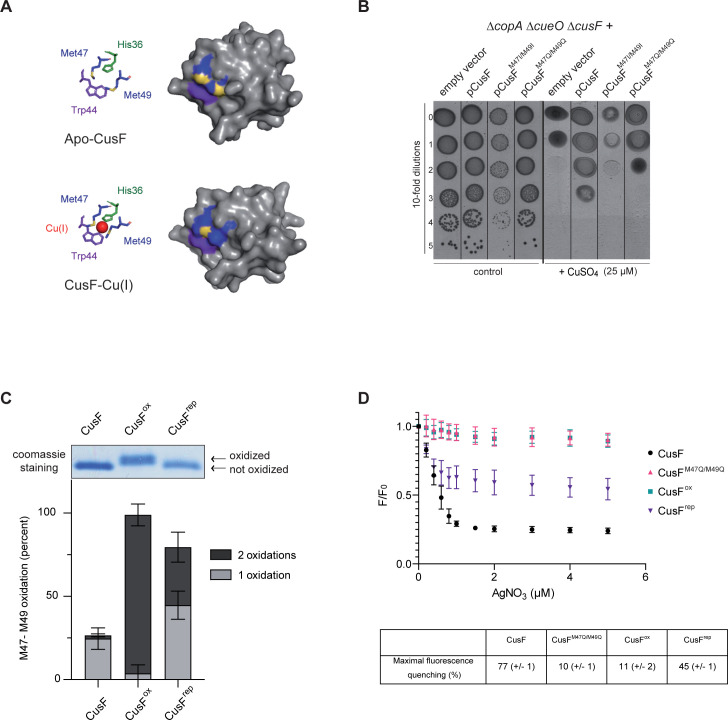
Methionine oxidation of CusF is deleterious. **A)** Aligned structures of the *E*. *coli* apo-CusF and CusF-Cu(I) adapted from PDB:1ZEQ and 2VB2 respectively [24,33] with stick and surface representations of CusF. Residues His 36 (green), Met 47, Met 49 (blue with sulphur atoms highlighted in yellow) and Trp44 (purple) are shown. The Cu(I) ion is shown in red. **B)** Plating efficiency of the Δ*copA* Δ*cueO* Δ*cusF* strain carrying empty vector, pCusF, pCusF^M47I/M49I^ or pCusF^M47Q/M49Q^ vectors onto M9 plates in the presence of CuSO_4_ (25 μM). The same protocol as described for Fig 2 was used, except plates contained ampicillin (50 μg/ml) and IPTG (50 μM). The images are representative of experiments carried out at least three times. **C)** Gel shift assay and mass spectrometry relative quantification by LFQ of the oxidation of Met47 and Met49. **D)** Silver binding analysed by quenching of intrinsic tryptophan fluorescence. Increasing concentrations of AgNO_3_ (0, 0.2, 0.4, 0.6, 0.8, 1, 1.5, 2, 3, 4, and 5 μM) were added to 1 μM CusF, CusF^M47Q/M49Q^, CusF^ox^ and CusF^rep^. The emission spectrum of CusF was recorded after each addition as described in the Materials and Methods. The integrated fluorescence peak (between 300 and 384 nm) in the presence of AgNO_3_ (F) was compared with the peak obtained in its absence (F_0_). The F/ F_0_ ratio was plotted against the concentration of AgNO_3_, after correction for the inner filter effect of AgNO_3_ measured on *N*-acetyltryptophanamide (NATA). The maximal fluorescence quenching for each variant of CusF was reported as a percentage in the table.
