# Supplementary material for: Periplasmic oxidized-protein repair during copper stress in E. coli: A focus on the metallochaperone CusF
Source: PLoS Genet. 2022 Jul 11;18(7):e1010180. doi: 10.1371/journal.pgen.1010180 (PMC9302797; doi:10.1371/journal.pgen.1010180)
Supplement: Attachment — Submitted filename: response to reviewers.pdf [file pgen.1010180.s002.pdf]

Responses to reviewers (comments in red / responses in black), modifications in the text are highlighted in yellow

Reviewer #1: This manuscript by Vergnes et al describes the Cu-based regulation of the MsrP methionine sulfoxide reductase by the CusSR two component system and an investigation of how MsrP interacts with elements of the E. coli CusCFBA Cu efflux pump. The authors demonstrate that MsrP is able to repair oxidative damage to Cu-binding methionine residues in CusF, which restores a Cu-sensitivity phenotype observed in the absence of CusF or MsrP.

This is a well written and presented manuscript, and the experiments presented clearly support the story that is being told. There are only some minor issues, including that some parts of the manuscript are a bit vague, in particular the initial investigation of the regulation of msrPQ by CusSR lacks key information, including how many promoters are present in the huiH-msrPQ (there is a small gap of ~ 109 bp between huiH and msrPQ – is there another promoter?), where the proposed CusR binding site is located, relative to the genes, and there is no information about the fact that CusR and HprR target the same sequence until the discussion section. However, it would be interesting to explore competitive effects of Cu and HOCl on the activation of huiH and msrP. Additionally unclear is why huiH is expressed much higher levels than msrPQ.

The authors have just published a paper that contains some of this information (Hajj etal 2022), and it would be good to make use of it here.

We thank the referee for the encouraging comments and the suggestions to improve our work. Indeed, in a recently published paper (El Hajj et al. 2022) we characterized the operonic organization of *hiuH*, *msrP* and *msrQ* genes. To our knowledge, there is only one promoter for this operon, downstream of *hiuH*. The gap between *hiuH* and *msrP* is particularly large and its molecular impact on *msrPQ* expression is currently under investigation. We suspect a post-transcriptional regulation that probably affects the stability of the *msrPQ* mRNA part. If this turns out to be true, then it would explain the difference in expression between *hiuH* and *msrPQ*. To make it easier for readers to understand, we have followed the advice of the referee by adding to Fig. 1 the operonic organization that includes the localization of the CusR and HprR binding sites (as previously described by Urano et al. 2007 and 2008).

Regarding the competitive effects of copper and HOCl, we thank the referee for these good suggestions. We had already carried out this type of experiment and have therefore added them to the results in the new version. These data are shown in the new Fig. 1E. Our results indicate that adding HOCl and copper together have the same impact on *msrP* expression as HOCl alone.

Line 137 now reads: “Interestingly, the HOCl/copper combination gave the same level of *msrP* expression as HOCl alone.”

C.N.R.S.- L.C.B. 31 Chemin Joseph Aiguier, 13402 Marseille CEDEX 20, France.
Tel. : (33) 4.91.16.40.77. Fax : (33) 4.91.71.89.14
Email : lcb-sec@imm.cnrs.fr

Specific comments:
Author summary:
L38-40 – unclear, what is the 'selective advantage' (which property of methionine?), and why would it necessitate the methionines being highly susceptible to oxidative damage (a similar passage is found in the discussion and this should be clarified in both instances.
We agree with the referee that this sentence was unclear. We changed line 39 to say: "Methionine emerges as a critical residue in copper trafficking proteins: however, its high affinity for metals is counterbalanced by its high susceptibility to oxidation."

Introduction:
L66 – please add some examples of which proteins involved in metal homeostasis were already identified as MsrP substrates.
As suggested by the referee, we have added examples of metal homeostasis proteins under the control of MsrP. Line 68 now reads: "proteomic studies have pinpointed processes including metal homeostasis (FecB, RcnB and ZnuA), under the supervision of MsrP [5]."

L 75-77 – why is Cu more toxic under anaerobic conditions, and if that is so, why is that not seen in the current study? The findings from the anaerobic exposure experiments presented in the results should be discussed in a detailed context on increased anaerobic Cu toxicity mechanisms and why there is no effect observed here.
We agree with the referee that we can be more explicit on this point; copper is more toxic under anaerobic conditions probably because it is predominantly present as Cu(I). Moreover, a new concept of copper toxicity during anaerobic condition, i.e protein aggregation (Zuili et al. 2022) has recently been published. Both notions were added to the new manuscript (lines 78-81). Moreover, we thank the referee 1 (and referee 3) for pointing out the lack of clarity in our results. Indeed we observed a difference in copper sensitivity under aerobic and anaerobic conditions, which is why we used less copper (1.5 µM) under anaerobic compared to aerobic conditions (5 µM). For improved clarity, we have added copper concentrations to the new panel Fig. 3A and we added a sentence to the text indicating copper concentrations (lines 162-165); now reads "While we observed a copper sensitivity of the Δ*copA* Δ*cueO* mutant was copper sensitive in the presence of 5 µM $CuSO_4$ under aerobic conditions, 1.5 µM $CuSO_4$ was sufficient to cause the same effects under anaerobic conditions due to the higher toxicity of copper in the absence of oxygen."

L103 – it would be good to at least mention what hype of enzyme huiH encodes early on, e.g. here. If msrPQ is regulated by CusSR, why was it not detected in the studies cited (28,29) that identified huiH as being CusR regulated. (also comments for l.115)
As requested, we modified the existing text by adding *hiuH* gene function early in the introduction (line 63). Authors of references 28 and 29 (Urano et al. 20015 and 2017) didn't know at the time that *hiuH* was part of the *msrPQ* operon and therefore never looked at *msrPQ* expression under copper treatment.

Results:

L116 – please update – '…investigated the role of copper…'
We have made the change as requested.

L137 - .. msrP is part of the CusCR regulon.. – Should that be msrPQ? And is this statemen based on the findings from the previous paragraph or referencing another study?
We agree with the referee that we can be more explicit here. In the new version, we now talk about the *hiuH-msrPQ* operon. This statement is based on Urano's paper and on our previous paragraph; they are now well identified (line 143).

C.N.R.S.- L.C.B. 31 Chemin Joseph Aiguier, 13402 Marseille CEDEX 20, France.
Tel. : (33) 4.91.16.40.77. Fax : (33) 4.91.71.89.14
Email : lcb-sec@imm.cnrs.fr

L149 – it might be good to clearly state that the copA, cueO and cusB triple mutant strains was made as part of this work.
The strain GG769 (ΔcopA ΔcueO ΔcusB::Kanr) is indeed part of this work as mentioned in the Table 1 “this study”. Because the copper hyper-sensitivity of a similar mutant (copA::kan ΔcueO ΔcusCFBA::cm) has already been described by the Rensing group (Grass and Rensing, 2001), we don’t think it is necessary to highlight the fact that the triple mutant was made as part of this work in the text.

L172 – ‘..whereas the copper sensitivity phenotype etc etc.’ Sentence unclear. Why would the empty vector affect the copper phenotype of mutant strains (Fig.4)? Are there any reasons why this might be the case.
We agree with the referee that this sentence was unclear. We didn’t know why the presence of an empty vector affected copper sensitivity. We can only speculate on the presence of antibiotics (needed to maintain plasmids) and IPTG (needed for gene expression), which can perturb copper stress by “scavenging” copper. Therefore, the second part of the sentence has been deleted in the new version.

L190 – please update – should that be ‘ with the sulfur atom accessible’?
We agree and we thank the referee for pointing out our mistake: as requested, we changed line 198 to say: “Analysis of the apo-CusF structure shows that Met47 and Met49 are exposed to the solvent, with the sulfur atoms accessible”

L195 ‘ mimetic’ – unclear, should that be mimic? Mimetic would be an adjective?
We agree with the referee, we changed line 204 to say: “Gln (Q), the latter mimicking Met oxidation”

L211 please update– Met47 and Met49 are part of the same peptide
We agree with the referee, we changed line 220 to say: “Met47 and Met49 are part of the same peptide and therefore, it was not possible to determine the oxidation level of each residue separately.”

L218 – please update – we took advantage of..
As requested, the correction has been carried out.

L223- please clarify – was the fluorescence quenching different or did it only appear to be different, i.e. probably was not different? The results are quite clear with a different of 65%?
We agree with the referee, we changed line 232 to say: “Compared to the native form of CusF, fluorescence quenching was strongly reduced with the oxidized form of CusF ($CusF^{ox}$): only a slight decrease in intrinsic fluorescence was observed, even at the highest $AgNO_3$ concentration tested (maximal fluorescence quenching = 11%).”

L230 “… that CusF oxidation disrupts…” – unclear. Your data suggests that the oxidation disrupts/ prevent Cu binding, however, I see no evidence that the mutations disrupt the protein, i.e. protein structure/ integrity?
We agree with the referee, we changed line 240 to say: “indicating that CusF oxidation affects the metal-binding capacity of the protein”. Moreover, as requested by reviewer #2, we performed circular dichroism spectroscopy analysis of the CusFox and CusFMtoQ mutant and we observed CD spectra similar to the one obtained with the native form of CusF (Fig. S1). This result shows that the CusFox is not disrupted in its structure/integrity.

L234 please update: ‘’.. the mutated M to Q forms of CusF in comparison..”
As requested, the correction has been carried out.

Discussion:

C.N.R.S.- L.C.B. 31 Chemin Joseph Aiguier, 13402 Marseille CEDEX 20, France.
Tel. : (33) 4.91.16.40.77. Fax : (33) 4.91.71.89.14
Email : lcb-sec@imm.cnrs.fr

L249/250 unclear – labile site – labile in what sense? Please add details. Why would the labile nature of the methionine be a selective advantage, and why would this necessarily have to be ' balanced' by high susceptibility to oxidation. Please clarify.
We agree with the referee that this sentence was unclear. We changed line 258 to say: " Methionine has emerged as a critical residue in copper trafficking proteins, providing binding sites that allow metal transfer. However its high affinity for metals is counterbalanced by its high susceptibility to oxidation."

L262 – should that be ' reduces met-O in CusF…)
As requested, the correction has been carried out.

L263-265 – sentence unclear, please reword
We agree with the referee, we changed line 273 to say: "Interestingly, the *msrPQ* and *cusCFBA* operons were both shown to be regulated by the CusSR TCS during copper stress. The existence of a common regulatory pathway for *msrP* and *cusF* reinforces the idea of a functional link between both proteins (Fig. 6)."

L269 – unclear , what are the different Cu defence systems that are expressed depending on growth conditions, and how do they relate to the work reported here? Why would the existence of these systems lead to the conclusion that MsrP maintains CopA and CueO under other (presumably not aerobic?) growth conditions. Please add sufficient detail to make this clear.
We agree with the referee that this sentence was unclear. Our idea was too vague and was pure speculation. For this reason, we deleted this sentence. However, we have kept the idea that CopA and CueO could be under the control of MsrP and this needs to be investigated in the future.
We changed line 275 to say: "However, the possibility that other components of the Cus pump are targeted by ROS/RCS cannot be excluded, as well as the CopA and CueO proteins, which also contain methionine-rich sites involved in copper binding. Testing this hypothesis will be a field of future research."

L273 Please update – 'copper participates in methionine…'
As requested, the correction has been carried out.

L288/289 – Where are the CusR/HprR boxes located relative to the huiH-msrPQ promoter? How many boxes and promoters are known for this gene region?
As for the first point, we have added a schematic operonic organization of *hiuH-msrPQ* in Fig. 1A. We hope it will be useful to the reader to better understand our work.

L300- …RclA prevents the formation…
As requested, the correction has been carried out.

Methods:
Throughout it is not fully clear when M9 or LB are used and why. Could that please be clarified?
We agree with the referee that throughout our work we used different media (LB; M9 and M9/CASA). This can be confusing and we need to clarify the medium used for each experiment. Initially we used LB to replicate the conditions found in the study describing induction of *hiuH* by copper (Urano et al. 2015), in order to facilitate comparisons. Then, it appeared to us that it would be better to work with a defined media (M9) in order to reduce variability. Media used in each experiment are now annotated in the text, legends and methods.

L401 – Why can 500 microM Cu-sulfate be used in M9/CASA medium, i.e. the concentration used for LB, rather than M9?
The referee is right we used 500 µM $CuSO_4$ in M9/CASA in Fig 1D-E. To our knowledge CASA is known to scavenge copper as LB compounds, so copper concentrations are higher in M9/CASA than in M9.

C.N.R.S.- L.C.B. 31 Chemin Joseph Aiguier, 13402 Marseille CEDEX 20, France.
Tel. : (33) 4.91.16.40.77. Fax : (33) 4.91.71.89.14
Email : lcb-sec@imm.cnrs.fr

L414 – what were the Cu concentrations used for this assay.
We thank the reviewer for this comment; we have added all the copper concentrations for each experiment. (line 427)

L442 – what concentration or protein to trypsin ratio was used here?
We thank the reviewer for this comment; we added the protein/trypsin ratio in line 458, by saying: “(to a final protease:protein ratio of 1:100)”

Figures:
Figure 4 – why does the presence of the empty vector change the Cu resistance phenotype of the strains in the absence of treatment? Is this experimental variation or was this observed in all replicates?
The referee is right: the presence of the empty vector changes the copper phenotype of the *copA cueO msrP* strain. We didn’t know why the presence of an empty vector affected copper sensitivity. We can only speculate on the presence of antibiotics (needed to maintain plasmid) and IPTG (needed for gene expression), which can perturb copper stress by “scavenging” copper. This effect was always observed in all replicates. This is also something we frequently see in oxidative stress experiments. Having talked about it with other microbiologists, we are not the only ones to have observed this kind of effect. Another proposed hypothesis would be the increase in the amount of DNA in the cell, but this has never really been tested.

Why were different concentrations of IPTG used for Figure 4 and Figure 5C
The IPTG concentration in Fig. 5B was decreased to 50 µM because expression of CusF (M/I) was very toxic with 100 µM IPTG. The data are included below (in absence of copper):

100 µM IPTG

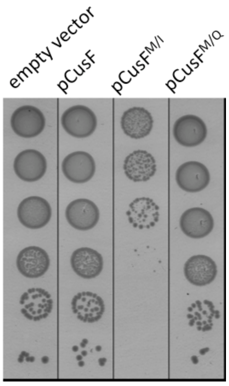

Table 3 – please include a reference of pJF119-EH where details of the plasmid can be looked up or add information about the plasmid backbone and the inserts that were added.
We thank the reviewer for this comment; we added to Table 3 the reference for pJF119-EH (Fürste et al. 1986 ; DOI: 10.1016/0378-1119(86)90358-6)

C.N.R.S.- L.C.B. 31 Chemin Joseph Aiguier, 13402 Marseille CEDEX 20, France.
Tel. : (33) 4.91.16.40.77. Fax : (33) 4.91.71.89.14
Email : lcb-sec@imm.cnrs.fr

Reviewer #2: This genetic and biochemical study thoroughly investigates the role of methionine oxidation in the metallo-chaperone CusF during copper stress. The authors found that the TCS CusRS activates the expression of the methionine sulfoxide reductase MsrPQ during copper stress, which appears to play a role for the functionality of the CusCFBA efflux pump under aerobic conditions. The authors further showed that CusF activity is impaired upon oxidation of two of its methionine residues, which can be rescued by MsrPQ. Overall, the study is well executed and includes all necessary controls. The manuscript is well-written and all conclusions stated by the authors are justified by the data presented. There is one major and a few minor comments the authors should address prior to publication:

Major comment:

For oxidation of CusF in vitro, the authors used 50 mM peroxide, which is a unphysiologically high concentration and likely leads to partial aggregation of CusF. This could potentially explain why the authors can only partially restore CusF activity in Fig. 5. If there is access to a circular dichroism spectrometer, the authors may want to examine the secondary structure of CusF(ox). It is likely that Copper stress generates ROS through the Fenton reaction, which could be quantified using ROS-specific fluorophores (i.e. H2DCFDA and others). This would give the authors an idea how much ROS is produced and that amount should be used to oxidize CusF.

We thank the referee for the encouraging comments and the suggestions to improve our work. The referee brings up an excellent point about the unphysiologically high concentration of hydrogen peroxide. We agree that 50 mM H_2O_2 is not biologically relevant; this in vitro treatment was only used to oxidize CusF.. In terms of kinetics, the oxidizing reactivity of Met residues with H_2O_2 is much lower than with HOCl ($2x10^2$ M^{-1} s^{-1} for H_2O_2 versus $3.4x10^7$ M^{-1} s^{-1} for HOCl), which is why the reaction requires a higher concentration of H_2O_2. During our work, we tried to oxidize CusF with HOCl and a concentration of 250 µM was enough to obtain Met-O in CusF, but unfortunately mass spectrometry analysis detects chlorination of His and Trp residues. This is why we kept the CusF-H_2O_2 treated form for our in vitro characterization.

In the non-cropped gel (below), we do not observe any aggregate of CusFox.

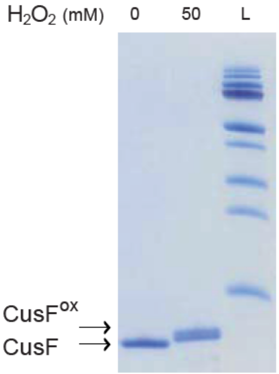

Concerning the secondary structure of CusF(ox), we thank the referee for this good suggestion. We performed circular dichroism spectroscopy analysis on CusF, CusF(ox) and the CusF M/Q variant. Our data showed no difference between the CD spectra obtained with these three forms, i.e. neither H_2O_2 oxidation nor M/Q mutations affected the overall conformation of the protein. These data are now included in the new Figure S1, and in the text line 245.

However, the referee is right: a local conformational change in CusFox could explain why MsrP partially restores CusF (Met-O content and metal binding). This could suggest the presence of an unknown periplasmic chaperone, which would work synergistically with MsrP. This hypothesis is reminiscent of the necessity for MsrA/B to work with the chaperone GroEL for repairing oxidized

C.N.R.S.- L.C.B. 31 Chemin Joseph Aiguier, 13402 Marseille CEDEX 20, France.
Tel. : (33) 4.91.16.40.77. Fax : (33) 4.91.71.89.14
Email : lcb-sec@imm.cnrs.fr

catalase in *Helicobacter pylori* (Mahawar et al., 2011). The characterization of this periplasmic chaperone is underway.

Copper stress generates HO° through the Fenton reaction in vivo, this has been demonstrated by the Imlay group using EPR (Macomber et al. 2007), which is more specific than generic ROS probes such as H2DCFDA. This is why it appeared to us that it was not necessary to redo this kind of analysis.

However, to answer the referee, using Calmodulin (CaM) as a protein model or CusF, we performed oxidation with copper and H_2O_2. Our result (below) shows that the Fenton reaction (100 to 500 µM H_2O_2 and 50 µM copper) leads to calmodulin and CusF oxidation. Unfortunately, we also observed protein degradation during H_2O_2/copper treatment (data below), making it impossible to use this kind of treatment to obtain CusFox.

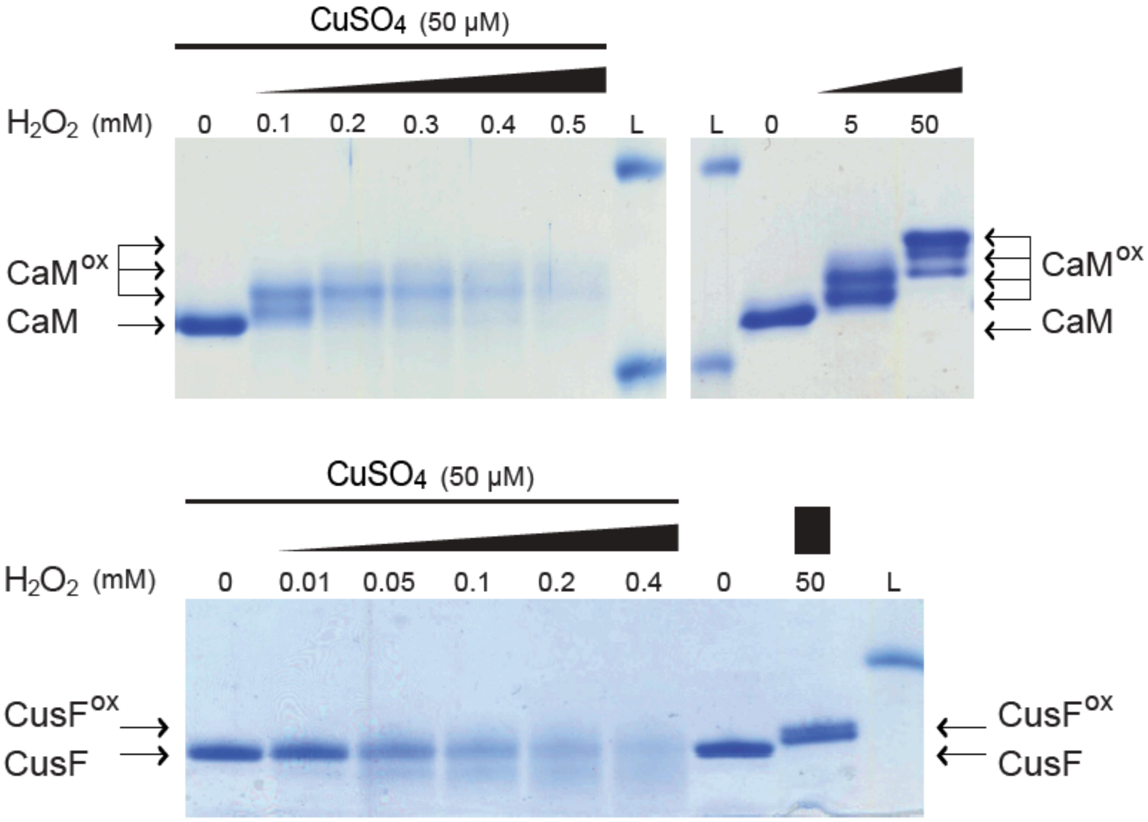

Minor comments:

1) Lines 78-87: the authors introduce the different copper -defense systems in E. coli. Given that they provide a schematic overview anyways in Fig 2B, they could refer to it here already.
We thank the reviewer for this comment, unfortunately in order to respect publishing guidelines it is impossible in the introduction to refer to a figure in the results.

C.N.R.S.- L.C.B. 31 Chemin Joseph Aiguier, 13402 Marseille CEDEX 20, France.
Tel. : (33) 4.91.16.40.77. Fax : (33) 4.91.71.89.14
Email : lcb-sec@imm.cnrs.fr

2) line 106: "... is induced during copper stress via CusSR. We then established a phenotypic..."
As requested, the correction has been carried out.

3) line 123: "....whether the TCS HprSR or CusSR regulate the expression ...."
As requested, the correction has been carried out.

4) The authors switch back and forth between different media (Fig. 1A: LB; Fig. 1C+D: M9/CASA; survival assays on plates: M9). What is their rationale for using different media?
We thank the reviewer for this comment. Initially we used LB to replicate the conditions found in the study describing induction of *hiuH* by copper (Urano et al. 2015), in order to facilitate comparisons. Then it occurred to us that it would be better to work with a defined media (M9) in order to reduce variability.

5) Generally, the authors should revise their materials & methods section, which lacks information essential for successful reproduction of the data. Among others, examples are:
a)What is is the concentration of casamino acids added to M9 media?
b) qRT-PCRL What was the start OD when copper was added to E. coli? How many hours/minutes was the strain exposed to copper?
c) line 433: What was the CusF concentration that was oxidized?
We thank the reviewer for this comment; we have revised the methods section by adding the missing information.

6) Comparison of Fig. 2C/3A (5 uM copper) and Fig 3B (25 uM copper): 5-fold higher copper concentrations in Fig. 3B don't seem to substantially affect the survival of the ΔcopAΔcueO strain. Do the authors have an explanation for the resistance?
We thank the referee for spotting our mistake: in Fig 3B we used 5 μM copper (and not 25). The correction has been carried out.

7) The study shows that overexpression of CusB and to some degree the entire cus operon is toxic for the cell. Do the authors have an explanation why particularly CusB has such a strong effect?
We thank the reviewer for this comment; we have no particular explanation, but it is known that the expression of a protein that is part of a complex, without its partners, can lead to this kind of toxicity. CusB is the periplasmic membrane fusion protein that links CusA in the inner and CusC in the outer membrane. Therefore, accumulation of CusB without CusA and CusC appears to be lethal for the cell.

C.N.R.S.- L.C.B. 31 Chemin Joseph Aiguier, 13402 Marseille CEDEX 20, France.
Tel. : (33) 4.91.16.40.77. Fax : (33) 4.91.71.89.14
Email : lcb-sec@imm.cnrs.fr

Reviewer #3: The authors investigated the interplay between CusFCBA-mediated copper resistance and the periplasmic oxidized protein repair system MsrPQ with MsrP being a methionine sulfoxide reductase. The respective operon was expressed under control of the two-component regulatory system of the Cus system, CusSR, and HprRS, which senses reactive chlorine species. MsrP is required for copper resistance in the absence of the copper-exporting P-type APTase Cop A and the periplasmic Cu(I) oxidase CueO under oxic conditions, if reactive oxygen species were not removed by catalase, but not under anoxic conditions. Expression of cusCFBA mollified this effect, and the periplasmic copper-binding protein CusF was mainly responsible. In the central experiment of this publication, the authors demonstrated that surface-exposed Met residues, which were also required for copper binding, were targets of oxidative stress, mutated or oxidized CusF was not longer able to bind the Cu(I)-proxy Ag(I), and, most important, that MsrP was indeed able to repair oxidized CusF to restore metal-binding activity of CusF. The interplay between copper, oxidative stress, MsrPQ and Cus is an important observation with broad interest to the metals field.

We thank the referee for the encouraging comments on the broad interest of our work.

1. Fig. 3. Copper is more toxic to E. coli under anoxic conditions, probably because it is predominantly present as Cu(I), than under oxic conditions. Fig. 3A, however, does not show any difference. Why? Secondly, "PBS" was no defined in the legend.
We thank the reviewer for this comment, which was also pointed out by referee 1. We do observe a difference in copper sensitivity under aerobic and anaerobic conditions, which is why we used less copper (1.5 µM) under anaerobic conditions compared to 5 µM under aerobic conditions. For improved clarity, we have added copper concentrations to the new panel in Fig. 3A and we have added a sentence to the text indicating copper concentrations (lines 162); now reads:" While we observed a copper sensitivity of the Δ*copA* Δ*cueO* mutant was copper sensitive in the presence of 5 µM $CuSO_4$ under aerobic conditions, 1.5 µM $CuSO_4$ was sufficient to cause the same effects under anaerobic conditions due to the higher toxicity of copper in the absence of oxygen."
As requested, PBS used to dilute catalase has been defined in the legend (line 547).

2. In the results, the headlines were not highlighted in any way, e.g. in blue as in the methods.
We are sorry but we don't understand this comment, the titles in the results were in blue like in the methods. We have kept this format in the new version.

3. The scientific style is unusual. Nearly every sentence starts with "We", "our" and reads more like a report of the last summer vacation for school and not like a scientific paper. It is customary that the authors step back behind their data. Moreover, simple past was not always used in the results. The data are nice, the description complete and concise, statistics done but I would strongly suggest to eliminate all "we", "us", "our" from the results part. It is OK for the last sentence of the introduction but not elsewhere.
We thank the referee for this comment on our data. We really regret that the referee did not like the scientific style of our manuscript. This way of writing is often found in scientific paper and allows the whole international community to understand the scientific results. Moreover, as his/her judgment is not shared by the two other referees (referee 1 «This is a well written »; referee 2 «The manuscript is well-written») we haven't changed everything, but we have taken his/her remark into consideration. We have reduced the number of "we" and "our" throughout the manuscript and we have checked the results presented in the results section are referred to in the past tense and made corrections when necessary.

C.N.R.S.- L.C.B. 31 Chemin Joseph Aiguier, 13402 Marseille CEDEX 20, France.
Tel. : (33) 4.91.16.40.77. Fax : (33) 4.91.71.89.14
Email : lcb-sec@imm.cnrs.fr
